# Résultats radio-anatomiques des prothèses totales du genou (à propos de 30 cas)

**DOI:** 10.11604/pamj.2015.20.414.6680

**Published:** 2015-04-27

**Authors:** Monsef El Abdi, Sidi Lamine Ouedraogo, Jonathan Bassinga, Abdelouahab Jaafar

**Affiliations:** 1 Service de Traumatologie Orthopédie « I », Hôpital Militaire Mohamed V, Rabat, Maroc

**Keywords:** Gonarthrose, prothèse, goniométrie, gonarthrosis, prosthesis, goniometry

## Abstract

La prothèse totale du genou correspond au remplacement prothétique de l'ensemble des compartiments fémoro-tibiaux et fémoro-patellaires. Ce travail est une étude rétrospective portant sur 26 patients pour un total de 30 PTG réalisées dans le service de chirurgie traumatologique et orthopédique de l'hôpital militaire d'instruction Mohammed V de janvier 2010 à décembre 2013 afin d’évaluer les résultats radio-anatomiques à l'aide d'un bilan radiologique « conventionnel » explorant le genou prothèsé dans les trois plans de l'espace et ainsi faire une comparaison avec les séries de la littérature.

## Introduction

Les arthropathies du genou sont des lésions fréquentes, elles sont dominées par les maladies arthrosiques et inflammatoires touchant le plus souvent Les deux genoux. Le traitement chirurgical ces affections a beaucoup progressé depuis les années 70 par le développement des prothèses totales du genou et par la qualité et la fiabilité de leurs résultats. [1]. Ses objectifs visent alors à lutter contre la douleur, à corriger les défauts mécaniques et à améliorer la fonction articulaire et la qualité de vie des patients. La prothèse totale du genou (PTG) est devenue une intervention fiable, reproductible, correspond au remplacement prothétique de l'ensemble des compartiments fémoro-tibiaux et fémoro-patellaire. L'indication doit en être cependant raisonnée et la prise en charge globale, fruit d'une collaboration étroite entre le rhumatologue, le chirurgien et le rééducateur fonctionnel [1]. L'information et la motivation du patient doivent être précisément établies et la nécessité d'une surveillance régulière ultérieure acquise. C'est à ce prix que les complications postopératoires compromettant le résultat fonctionnel seront au mieux évitées. L′objectif de cette étude rétrospective que nous rapportons est d’évaluer les résultats radiologiques des patients qui ont bénéficié d'une PTG.

## Méthodes

Nous rapportons une étude rétrospective portant sur 30 prothèses totales du genou chez 26 patients colligées au sein du service de chirurgie traumatologique et orthopédique « 1 » de l'hôpital militaire d'instruction Mohammed V de Rabat, étalée sur 4 ans, entre 2010 et 2013. Les méthodes d’étude ont été basées sur l'exploitation des dossiers médicaux avec recueil des données surtout radiologiques, afin de comparer nos résultats avec ceux de la littérature. L’évaluation des résultats radiologiques a été réalisée sur des radiographies standards (face en charge, en Shuss, profil stricte à 30°, défilé fémoro-patellaire 30° et 60°), et un pangonogramme en charge avec appui bipodal. Les principaux critères radiologiques étudiés sont: l'axe mécanique du membre inferieur (angle HKA) pré et post opératoire; La hauteur rotulienne (indice de Caton et Deschamps) pré et post opératoire; La pente tibiale (la méthode de Moore HARVEY) pré et post opératoire; Le type d’équipement; La qualité du ciment Le resurfaçage rotulien; Le centrage rotulien. L'usure cartilagineuse fémoro-tibiale a été appréciée selon la classification d'AHLBACK. L'analyse statistique a été effectuée par le logiciel SSPS 10 (le seuil de significativité « p » a été fixé à 0,05).

## Résultats

L’âge de nos patients variait entre 49 et 84 ans, avec une moyenne de 67,8±9,12 ans. 77% d'entre eux avaient un âge supérieur à 60 ans. On note une prédominance féminine de 63,3% avec 19 femmes pour 11 hommes. Le côté droit était atteint dans 60% (18 PTG) des cas contre 40% (12 PTG) pour le côté gauche. Tous nos malades ont été opéré pour gonarthrose qui était primitive dans 24 cas soit 80%, et secondaire (post-traumatique, atteintes inflammatoires) dans 6 cas soit 20%. Le délai de consultation variait entre 06 mois et 5 ans, dont 90% des malades ont consulté 1 an après le début des symptômes. Le séjour hospitalier était en moyenne de 10 jours avec des extrêmes de 5 à 20 jours. L'usure fémoro-tibiale selon la classification des gonarthroses d'Ahlbäck (1968) modifiée par H.Dejour (1991): le stade 1 était présent chez 1 cas soit 3,3%, le stade 2 (7 cas soit 23,3%), le stade 3 (16 cas soit 53,4%), le stade 4 (6 cas soit 20%). L'usure fémoro-patellaire: externe (4 cas soit 13%), globale (23 cas soit 77%), absente (3 cas soit 10%). La voie d'abord para-patellaire interne a été utilisé dans 26 cas (87%), 4 cas (13%) ont été opérés par voie para-patellaire externe. L'angle moyen HKA préopératoire était de 171,8 (161-185), et HKA postopératoire était de 176,4 (168-187). On note une normocorrection HKA = 180° ± 3° dans 15 cas soit (50%), et une hypocorrection HKA ≤ 176° dans 15 cas soit (50%). La rotule était centrée dans 29 cas (soit 97,7%), un seul cas avait subluxation externe. La hauteur moyenne de la rotule était de 1,06 [0,85-1,21], avec une moyenne en préotopératoire de 0,77, et en postopératoire de 1,017. La valeur moyenne de la pente tibiale en pré et postopératoire était de 7° ± 1,8° et 5,83° ± 1,53° respectivement. Le resurfaçage rotulien a été réalisé dans 10 cas, soit 33,33%. Toutes les prothèses ont été ont été cimentées. Nous avons utilisé trois marques de prothèse tri-compartimentale postéro-stabilisée à plateau mobile (Tableau 1).

**Tableau 1:** Résultats des différentes caractéristiques de l’étude statistique (valeur moyenne de l'ensemble des patients

Caractéristiques		valeurs moyennes (n = 30)
Age en années		67,80± 9,12
Sexe	Féminin	19 (63,3%)
	Masculin	11 (36,7%)
Côté	Droit	18 (60%)
	Gauche	12 (40%)
HKA	préopératoire	171[169-174,2]
	postopératoire	177[174-179]
Indice de Caton		1,06[0,85-1,21]
Pente tibiale	préopératoire	7[5-8]
	postopératoire	5,83 ± 1,53
Type de Prothèse	Zimmer	17 (56,6%)
	Score	2 (6,7%)
	Lépine	11 (36,7%)
Resurfaçage rotulien	Non	20 (66,7%)
	Oui	10 (33,3%)
Centrage rotulien	Aligné	29 (96,7%)
	Subluxé ou luxé	1 (3,3%)
Score Ahlback	Satde 2	1 (3,3%)
	Satde 3	7 (23,3%)
	Satde 4	16 (53,4%)
	Satde 5	6 (20%)

## Discussion

L’âge moyen dans notre série était de 67,8±9,12 ans avec des extrêmes de 49 à 84 ans, on note également que l'indication des PTG s’étend à des patients de plus en plus jeunes et cela peut être expliquer par l'arthropathie inflammatoire, cela est proche de la série de **BRIARD** [2] avec un âge moyen de 68 ans et des extrêmes allant de 43 à 77 ans, tandis que dans la série de **NEYRET** [3] l’âge moyen était de 76,6 avec des extrêmes de 70 à 87 ans. Ainsi, les auteurs ne sont pas arrivés à déterminer le rôle de l’âge dans l’évolution des arthropathies du genou. La majorité des séries font état d′une prédominance féminine [3–7], les résultats de notre série vont dans le sens de cette constatation. La quasi-totalité de nos malades consultent à un stade tardif, souvent adressés par des rhumatologues et des médecins généralistes. Ce délai peut être expliqué par le caractère discret des symptômes. Le séjour hospitalier était en moyenne de 10 jours avec des extrêmes de 5 jours à 20 jours. Ce délai relativement prolongé est expliqué par le début de la rééducation au sein du service et la surveillance des complications post opératoires immédiates. La gonarthrose revêt de différentes formes étiologiques [8]. Dans notre série nous avons trouvé 24 cas soit 80% d'arthrose primitive, et 6 cas soit 20% d'arthrose secondaire. Le traitement chirurgical par prothèse du genou est utilisé en pratique courante clinique depuis environ 30 ans, tout d'abord de façon épisodique et en utilisant uniquement des prothèses à charnières dont les résultats ont été médiocres. Actuellement, l'utilisation des prothèses à glissement et surtout le développement des techniques de positionnement très rigoureuses et précises ont transformé les résultats de ces prothèses. Toutes nos prothèses étaient cimentées, postéro-stabilisées à plateau mobile sans conservation des ligaments croisés. D'autres études les auteurs ont utilisé des équipements prothétiques différents avec ou sans ciment [3–7, 9–12]. Dans notre série la quasi-totalité des genoux (26 PTG) ont été opérés par voie d'abord para-patellaire interne. Cela concorde avec la littérature [10–12]. L'abord médial ou latéral est lié à l'importance de la déformation frontale préopératoire et à la rétraction des parties molles. L’évaluation de la correction par le calcul de l'axe du membre (angle HKA) est, avec l’évaluation de la pente tibiale ainsi que le centrage rotulien, l'un des trois points essentiels de cette étude. Dans notre série, nous avons objectivé une différence statistiquement significative entre HKA pré-opératoire et HKA post-opératoire avec un degré de significativité p = 0,002. ces résultats sont comparables a ceux d′autres auteurs [13–16] (Tableau 2). Dans toutes les séries étudiées [13, 17–19], la pente tibiale pré-opératoire est peu différente de celle post-opératoire, cette tendance est confirmée dans notre série avec une différence de 0,5° qui est statistiquement significative avec un pTableau 3). Dans la série de T.Ammari [13] (26 PTG) la hauteur rotulienne pré-opératoire était peu différente de celle post-opératoire, alors que dans notre série la différence est plus notable. Cette différence n'est pas significative (Tableau 4). Les complications patellaires représentent une principale cause d’échec dans les arthroplasties totales de genou [20]. Dans notre série nous n'avons noté qu'un seul cas de subluxation latérale, ce qui représente un excellent résultat comparativement aux autres séries [21–25].

**Tableau 2:** Tableau comparatif de la correction angulaire selon les différentes séries

	Pré op	Post op	P
T.Ammari [13]	175,5°	178,8°	0,005
B.Zniber [14]	178,1°	179,5°	0,060
R.S.Laskin [15]	176,1°	178,9°	0,006
Hajime Yakamana [16]	173,3°	174,7°	0,005
**Notre série**	**171,8°**	**176,4°**	**0,002**

**Tableau 3:** Tableau comparatif de la pente tibiale pré et post-opératoire dans les différentes séries

Série	N	Pente tibiale		
		Pré Op	Post Op	P
Brouwer [17]	14	9,5	11,9	0,002
El Ammari [13]	41	4,2	6,3	0,06
El Azab [18]	53	6,4	8,4	0,003
A Ducat [19]		5,6	6,2	0,001
**Notre série**	**30**	**6,6**	**7,1**	**<0,001**

**Tableau 4:** Tableau comparatif de l'indice de Caton et Deschamps de notre série avec celle de T.Ammari

	Indice de Caton et Deschamps		
	Pré Op	Post Op	P
**T.Ammari** [13]	0,77	0,81	**0,3**
**Notre série**	0,77	1,06 [0,85-1,21]	**0,23**

## Conclusion

La prothèse totale du genou occupe une place importante dans le traitement des arthropathies du genou évoluées ou étendues à plusieurs compartiments où tout procédé de conservation parait dépassé. Les résultats dépendent d'une part d'une bonne planification pré opératoire et, d'un geste technique irréprochable, d'autre part d'une rééducation postopératoire efficace et une motivation réelle du patient.
